# Neonatal and Infantile Immune Responses to Encapsulated Bacteria and Conjugate Vaccines

**DOI:** 10.1155/2008/628963

**Published:** 2008-09-23

**Authors:** Peter Klein Klouwenberg, Louis Bont

**Affiliations:** Department of Pediatric Infectious Diseases and Immunology, Wilhelmina Children's Hospital, University Medical Centre, Room KE4.133.1, P.O. Box 85090, 3508 AB Utrecht, The Netherlands

## Abstract

Encapsulated bacteria are responsible for the majority of mortality among neonates and infants. The major components on the surface of these bacteria are polysaccharides which are important virulence factors. Immunity against these components protects against disease. However, most of the polysaccharides are thymus-independent (TI)-2 antigens which induce an inadequate immune response in neonates and infants. The mechanisms that are thought to play a role in the unresponsiveness of this age group to TI-2 stimuli will be discussed. The lack of immune response may be overcome by conjugating the polysaccharides to a carrier protein. This transforms bacterial polysaccharides from a TI-2 antigen into a thymus-dependent (TD) antigen, thereby inducing an immune response and immunological memory in neonates and infants. Such conjugated vaccines have been shown to be effective against the most common causes of invasive disease caused by encapsulated bacteria in neonates and children. These and several other approaches in current vaccine development will be discussed.

## 1. INTRODUCTION

Globally, more than 2.5 million infants die every year from bacteremia, respiratory, and diarrhoeal diseases [1]. A limited number of viral and bacterial pathogens are responsible for this burden of disease among neonates and infants. A study by the WHO has identified that the most pathogenic bacteria are encapsulated bacteria, such as *Streptococcus pneumoniae*, *Streptococcus pyogenes, Staphylococcus aureus*, *Escherichia coli, Neisseria* *meningitidis*, and *Haemophilus* *influenza* [2]. The capsule around these pathogens is formed by a polysaccharide coating. The immunologic advantage of this coating is evasion of phagocyte killing, as the coating blocks complement binding and opsonization. This can be overcome by C-reactive protein (CRP) binding [3] and the production of antibodies against the polysaccharide [4]. This immune response confers protection against disease. However, especially the young [5], but also the elderly [6], have a weak immunological response to these encapsulated bacteria due to the thymus independent (TI) nature of these bacteria.

The humoral immune response to antigens can be divided into thymus-dependent (TD) and TI-responses [7] (see Table 1). TD antigens consist of soluble proteins or peptides and associate with major histocompatibility complex (MHC) molecules at the surface of an antigen presenting cell (APC), thus allowing the APC to interact with CD4+ T cells. In contrast to TD antigens, TI antigens do not require T-cells to induce an immune response [8]. Therefore, TI antigens do not or poorly induce an immunological memory. The antibodies that are produced are primarily of the IgM isotype and in lesser quantities IgG2 [7]. The TI antigens are further divided into two categories based on their interaction with B cells: type 1 (TI-1) and type 2 (TI-2) antigens [7–9]. TI-1 antigens induce proliferation and differentiation of B lymphocytes and induce immune responses in adults, but also in neonates [5, 10]. TI-2 antigens on the other hand induce a limited immune response in children below two years of age, but older children and adults react to TI-2 antigens with the formation of sufficient antibody production by activated B-cells. TI-1 antigens include lipopolysaccharides, which is part of the Gram-negative bacteria cell wall [6]. Therefore, immune responses to Gram-negative bacteria are relatively sufficient in neonates and infants compared to encapsulated bacteria, but still below levels of those of adults [10, 11]. TI-2 antigens are bacterial polysaccharides from encapsulated bacteria such as most *S. pneumonia* serotypes, *N.* *meningitidis*, and *H. influenza* [12]. Infection with these bacteria results in a reduced immunological response in neonates and therefore they are at risk [5]. The polysaccharides of some other bacteria such as *S.* *pneumoniae* serotype 3, however, are not classified as TI-2 antigens.

The global use of effective vaccines directed against encapsulated polysaccharide pathogens would reduce the morbidity and mortality among newborns and infants significantly [13]. In poor resource countries medical care strikingly decreases after the first year of life, necessitating the development of effective infant immunization programs [14]. The challenge for early life immunization is to induce sustained protection circumventing immaturity of the immune system to TI-2 antigens [15]. Neonatal and infantile antibody responses to vaccines are of short duration and decline rapidly within a few months [16]. This may be associated with a resurgence of vulnerability to infection, requiring the administration of repeated vaccinations already in the second year of life. A better understanding of the neonatal response to polysaccharide antigens may lead to the development of improved vaccines. This review will explore the neonatal immune response on polysaccharide antigens and on polysaccharide protein conjugates. Furthermore, the efficacy and drawbacks of the three main polysaccharide-conjugate vaccines will be discussed.

## 2. NEONATAL IMMUNE RESPONSES TO POLYSACCHARIDES

There is a marked limitation in neonatal and infantile antibody responses to most, but not all, bacterial capsular polysaccharides [5]. The mechanisms that are thought to account for the partial unresponsiveness of neonates to TI-2 stimuli will be reviewed in the next paragraphs.

### 2.1. B cell immaturity

Polysaccharide antigens localize preferentially to the marginal-zone (MZ) B cells, found only in the spleen. These B cells are present in low numbers at birth and the development is deficient in neonates [17]. MZ B cells with adult features appear after 2 years of life and coincide with the ability to induce an immune response to polysaccharides. Children under two years of age have a quantitative defect in IgG2 and IgG4 isotypes [18]. Although other isotypes reach adult levels by two to three years of age, IgG2 appears much later in development, and adult levels of this subclass are not reached until 5–10 years of age [19]. As the IgG2 isotype is considered as the most effective immunoglobulin against some polysaccharides [20], the susceptibility of neonates and infants might be due to the defect in immunoglobulin production. Furthermore, a dysfunctional spleen or splenectomy increases the risk of infection by encapsulated bacteria, such as *S. pneumoniae* and requires antibiotic prophylaxis [21] or vaccination [22].

The possibility that B cell immaturity in the MZ B cells might cause the reduced TI-2 antigen response was first observed in mice by Mosier et al. [23]. With the development of the murine immune system, B cells change from IgM^hi^IgD^lo^ to IgM^lo^IgD^hi^ and the response to TI-2 antigens coincided with the appearance of IgD, which takes about one to two weeks in mice [24]. Furthermore, it was shown that mice with an X-linked immune deficiency (CBA/N mice) resulting in B cells that phenotypically resemble neonatal B-cells are unable to respond to TI-2 antigens [23]. The murine MZ expresses the specific intercellular adhesion molecule-grabbing nonintegrin receptor 1 (SIGNR1) which plays a relevant role in the immune response against encapsulated bacteria [25, 26], but its human homologue has not yet been found.

### 2.2. CD21 and complement

The immune response to polysaccharides is initiated when polysaccharides activate complement factor C3d via the alternative pathway [27]. The resulting polysaccharide-complement complex subsequently localizes in MZ B cells expressing CD21 (complement receptor 2) [27, 28]. Neonatal and infantile B cells have low expression of CD21 which explains the inadequate response to polysaccharides [17, 28]. Interestingly, the increase of CD21 that occurs during development coincides with the response to polysaccharides. Furthermore, neonates have relatively low levels of complement [29]. Therefore, in early age, CD21 cannot bind the polysaccharide-C3d complex sufficiently and antibodies are not produced. Conjugate vaccines, however, are complement independent and induce an antibody response in neonates and young children, which implies that MZ B cells are not needed for this response.

In a murine model, Breukels et al. [30] showed that polysaccharide-conjugates localize in the splenic MZ of neonatal mice without obvious relation to MZ B cells. Furthermore, it was shown in adult mice that the antibody response to polysaccharides is absent after cobra venom factor (CVF) treatment, which depletes complement.

### 2.3. T lymphocyte and cytokine defects

In spite of their name, in mice TI-antigens need the assistance of unspecific T-cells and cytokines [31]. In neonatal mice these signals are absent or diminished and B cells activated by TI-antigens do therefore not differentiate. Neonatal accessory cells are deficient in interleukin (IL)-1 and tumor necrosis factor (TNF)-*α*, which are important for TI-2 responses [32, 33]. Supplementation of a mouse model with these cytokines enabled neonatal B cells to clonally expand and differentiate [6, 32]. The reduced IL-1 and -6 production in neonatal mice by macrophages seems to be related to a decreased level of several TLR’s (TLR2, TLR4) [34, 35]. Also the increased activity of p38 MAPK, a mitogen-activated protein kinase that is involved in the TLR intracellular signaling pathway, seems to play a pivotal role in the unique cytokine phenotype of the neonatal macrophages [35]. The identity of T cells involved in antibody responses to TI-2 antigens is still unknown. Kobrynski et al. [31] measured IgG antibody production after immunization with pneumococcal polysaccharides in mice with disruptions in selected genes of the T cell pathway. Nonclassical MHC class I-like CD1 molecules and a subset of MHC class I-dependent CD8+ cells were found to be essential for antibody responses to TI-2 polysaccharides.

### 2.4. Other hypotheses

Several other explanations have been proposed and rejected. A specific B cell subset, B-1, is thought to be important for TI-2 responses since certain TI-2 specificities are found in this subset. However, neonates have concentrations of B1-cells comparable to adults and it seems unlikely that this is the cause of reduced TI-2 responses [6]. Another view was based on the increased susceptibility of immature B cells to tolerance induction [36]. Lastly, it was thought that TI-2 antigens cause a relative increase in suppressor T cells compared to amplifier T cells in a murine model, which reflects an imbalance between Th1 and Th2, in favor of Th1 cells. However, this is unsatisfactory since the current data suggest a Th1 deficiency in neonates [6].

## 3. POLYSACCHARIDE CONJUGATE VACCINES

**Figure 1:**
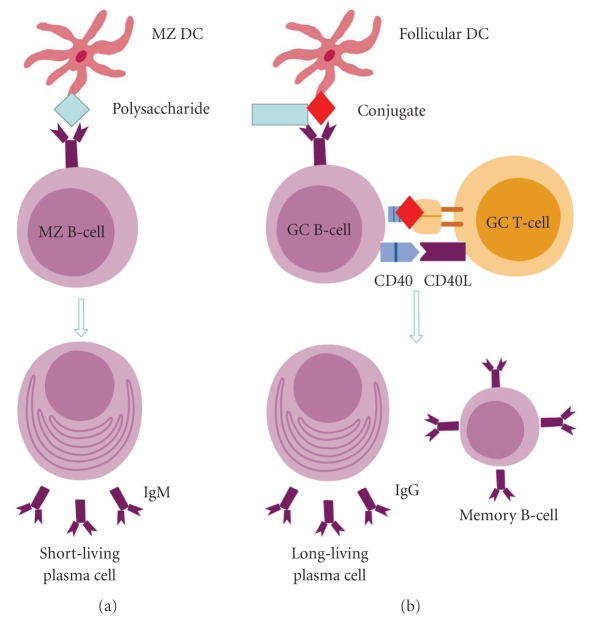
Schematic diagram of mechanism of action of PS and PS-protein conjugate vaccines. (a) Polysaccharide vaccines stimulate marginal zone B-cells which will proliferate in loco and differentiate into short-living plasma cells, which are responsible for the rapid release of low-affinity antibodies and thus first-line defence against the pathogen; (b) conjugate vaccines stimulate B-blasts that will migrate into the germinal centre, where they proliferate, undergo somatic hypermutations and isotype class switch, and differentiate into either long-living plasma cells (that produce high-affinity antibodies) and memory B cells. Abbreviations: CD40L (CD40 ligand), GC (germinal centre), MZ (marginal zone), DC (dendritic cell), MZ DC (marginal zone dendritic cell).

The impaired neonatal and infantile immune response to polysaccharide vaccines can be circumvented by conjugating the polysaccharide to a protein carrier [37–39], based on the old dogma that haptens when attached to a protein carrier can induce an immune response. The mechanism by which a polysaccharide-protein conjugate vaccine acts is depicted in Figure 1. Today, all current polysaccharide vaccines registered for children below age two are conjugated vaccines. Conjugates transform bacterial polysaccharides from a TI-2 antigen into a TD-antigen and thereby induce an immune response and immunological memory in neonates [40], however not to the extend as in adults [4]. Several factors which are related especially to immunizations might contribute to a suboptimal response to conjugate vaccines. Firstly, the route of immunization determines the recall response to polysaccharides in mice that have been vaccinated with a conjugate as neonates [41]. Secondly, the choice of type of protein conjugate is important and determines the amount of IgG antibody response [41]. Lastly, in neonatal mice there is a Th-2 skew which leads to a predominantly IgG1 response and impaired IgG2a antibody formation [4], the latter thought to be more protective against encapsulated bacteria [6].

### 3.1. Haemophilus influenza B conjugate vaccine

Several *Haemophilus influenza B* (HiB) conjugate vaccines were introduced in the early 1990s. The reported efficacy against meningitis and epiglottitis ranged from 94% in children of 2-3 years of age to 99% for infants below 1 year of age [42]. In Brazil, the conjugate vaccine led to a decrease in HiB meningitis 2.39 to 0.06 cases per 100 000 population (98%) overall, and from 60.9 to 3.1 cases per 100 000 population (95%) in children <1 year of age, five years after the introduction of the vaccine [43]. Furthermore, the HiB conjugate vaccines reduced carriage of HiB [44] and probably led to lower transmission rates to children who lacked protective antibodies.

### 3.2. Neisseria meningitidis conjugate vaccine

Purified polysaccharides from *N. menigitidis* serogroups A, C, W135, and Y are available vaccine products and elicit antibody responses with no memory function, with the exception of serogroup A polysaccharide which induces a marginal antibody response also in infants [45]. The serogroup C polysaccharide is not immunogenic in children below 2 years of age, and development of antibody titers is slow [46]. Conjugates have been developed using the same principles as for HiB. The type A and C conjugate vaccines are safe and well tolerated in infants and young children [47]. In Spain, the meningococcal C vaccine (MCC) was effective in 98% of infants vaccinated at two, four, and six months of age and 99% in those vaccinated after seven months of age [48]. However, the vaccine effectiveness fell after the first year, especially in those vaccinated as infants. In England, the estimated effectiveness was 66% in infants vaccinated at two, three, and four months of age and 83% in those vaccinated after seven months of age [16]. It fell to low levels after one year in those vaccinated in the first year of life. A vaccine against type B (MenB), which is a common cause of meningococcal disease, is presently unavailable [49, 50]. However, unpublished data provide a source of optimism to develop a safe and effective containing recombinant outer membrane surface proteins of MenB vaccine from the *N. meningitidis* strain NZ98/254. This investigational vaccine against MenB induced protective immune responses against 3 strains after the third dose (Miller et al. abstract 133, Annual Meeting of the European Society of Paediatric Infectious Diseases (ESPID, 2008)). Results from Phase 3 RCTs are expected during the coming years.

### 3.3. Streptococcus pneumoniae conjugate vaccine

Diseases caused by pneumococci include pneumonia, meningitis, otitis media, sinusitis, and bronchitis. Pneumococcal vaccines that are effective in infants have been welcomed as resistance against growing commonly used antibiotics [51]. An unconjugated 23-valent vaccine is registered for children over two years of age but is ineffective in younger children [52]. A 7-valent polysaccharide-protein conjugate vaccine (PCV-7) has been introduced for use in children below the age of 2 years. Serotypes included in PCV-7 cover 65–80% of serotypes that cause invasive pneumococcal infections [53]. Other conjugate vaccines with wider serotype coverage, including a 10-valent vaccine and a 13-valent vaccine, are in the late stage of development [54, 55]. A 94% decrease in vaccine type invasive pneumococcal disease in children with a coverage rate of just 68% was reported [56]. The number of all-cause pneumonia admission rates has declined by 39% for children younger than two years in the USA from 2000–2004 [57] and admissions due to pneumococcal meningitis were reduced by 66% in the same period [58]. A significant decrease was also seen in the unvaccinated groups as a result of herd immunity due to decreased transmission from vaccinated children to unvaccinated contacts [56]. The protective efficacy against acute otitis media however has been relatively modest. In a Finnish study, the efficacy against confirmed otitis media was 34% and the overall efficacy against otitis media regardless of cause was only 6% [59].

### 3.4. Other conjugate vaccines

Several other polysaccharide pathogens are under investigation. Group B streptococci are the major cause of meningitis and sepsis in neonates. In animal studies, group B streptococcal conjugate vaccines have shown to be able to induce protective antibodies [60]. Similar attempts have been made to develop immunogenic and safe vaccines against the Vi polysaccharide of *S. typhi* [61] and polysaccharides and LPS of *E. coli* [62] as well as *S. aureus* [63].

## 4. DRAWBACKS OF CONJUGATE VACCINES

As described above, polysaccharide-conjugate vaccines are effective in children under the age of two years. However, conjugate vaccines are only available for *H. influenza type B*, some meningococcal subtypes, and recently for a limited number of subtypes of *S. pneumoniae*. Vaccine failure and rise of nonserotype bacteria have been noticed and require continuous attention [64]. There is evidence that coadministration with other vaccines may impair effectiveness of the vaccinations [65]. Finally, conjugate vaccines are expensive, which does not allow for global use of these vaccines [53].

### 4.1. Haemophilus influenza B conjugate vaccine

A decade after its introduction in the UK, the first conjugate vaccine, HiB, became less effective and vaccine failures were seen [66]. Two explanations have been given for this effect. First, after early age immunization antibody levels are sufficient for protection but drop over the following years, sometimes to levels that are not considered protective. This was explained by a reduction in either the number or the quality of memory B cells induced by immunization or a loss of avidity in matured B cells prior to disease onset following defective priming [67]. The low titers observed in the UK may have been exacerbated by the loss of “natural boosting” associated with a reduction in carriage. Reboosting might be the solution [67]. The other important factor contributing to the reduced immune response to HiB-conjugate-vaccine is the current coadministration with other vaccines. Reduced antibody responses to HiB conjugates have been documented using acellular pertussis/HiB [65], DTaP-HiB [68], and MCC/HiB combinations [69], but not in other studies [70, 71]. The impaired immune responses were increased by accelerated immunization schedules such as that in the UK [72]. The precise immunological mechanism responsible for the excess of vaccine failures following the combination vaccine is not known.

### 4.2. Neisseria meningitidis conjugate vaccine

The meningococcal serogroup B polysaccharide is poorly immunogenic in man [73]. The development of an effective vaccine against *N. meningitidis* serogroup B is complicated by the inability of this polysaccharide to induce a significant antibody response [73], even when conjugated to a carrier protein [74]. However, unpublished data show promising results (Miller et al. abstract 133, Annual Meeting of the European Society of Paediatric Infectious Diseases (ESPID, 2008)). In Spain and the UK, the MCC vaccine effectiveness fell after the first year, especially in those vaccinated as infants [16, 48]. These data suggest that the protection given by MCC vaccine may be age dependent and that children vaccinated at an older age may have greater and longer-lasting protection than those vaccinated as infants. This suggests that protection may be more reliant on circulating antibodies at the time of exposure than on the ability to mount a booster response [75]. A recent study found that the immune response and length of protection were dependent on the formation of a large germinal center one month after primary immunization with the MCC vaccine [76]. One third of infants in this study produced a very low number of memory B cells after the initial immunization and did not maintain protective antibody levels by one year of age. In these children, the germinal center was underdeveloped. Understanding of the factors that determine the production of these germinal centers could lead to improved conjugate vaccines. Until then, booster doses of MCC may be required in order to extend the duration of protection offered by the vaccine.

### 4.3. Streptococcus pneumoniae conjugate vaccine

A major drawback of pneumococcal conjugate vaccines is that the serotypes included in PCV-7 cover only 65–80% of serotypes that cause invasive pneumococcal infections [53]. Efforts to include more subtypes in a conjugate vaccine prove to be very complicated and costly. Furthermore, it has been shown that combination of PCV-7 with other vaccines can lead to reduced immune responses. The response to hepatitis B vaccine was nonsignificantly reduced with concomitant administration with PCV-7 [77]. Another problem is that the PCV-7 vaccine replaces disease by nonvaccine serotypes especially 19A [78] and 16F [59]. A recent study in Alaska, where routine vaccination in children has started in 1999–2000, showed an increase in invasive pneumococcal disease rate caused by nonvaccine serotypes of 140% compared with the prevaccine period [64]. In the first three years after introduction of the PCV-7 vaccine, there was a 96% decrease in heptavalent vaccine serotype disease. This led to a decrease in overall invasive pneumococcal disease of 67% in Alaskan children younger than 2 years (from 403.2 per 100 000 in 1995–2000 to 134.3 per 100 000 per year in 2001–2003) but to an 82% increase in invasive disease in the following years to 244.6/100 000. Serotype 19A accounted for 28% of invasive pneumococcal disease among Alaska children younger than 2 years during 2004–2006. There was no significant increase in disease due to nonvaccine serotypes in nonnative Alaskan children younger than 2 years [64]. This emphasizes the importance of continuing surveillance and development of expanded valency vaccines. The question remains whether this serotype shift leads to increased morbidity and mortality rates as these nonvaccine types are typically less pathogenic. Other limitations are a modest effect on nasopharyngeal colonization [79], cost (US*$* 32 000–166 000 per life-year saved) [53], and difficulties in production that have led to shortages.

## 5. NEW DEVELOPMENTS

The currently registered conjugated polysaccharide vaccines have been developed based upon the principle that CD4 T cell recruitment is necessary for the activation of the infant B cell immune response [8]. The underlying thought was to promote a transformation of the neonatal immune response from a TI one to a TD one by conjugating the polysaccharides to immunogenic carrier proteins. The neonatal immune response to TD-antigens has been shown to be better than the response to TI-2 antigens but did not reach levels seen in healthy adults [4]. One of the most important factors determining the neonatal immune response to conjugate vaccines seems to the type of carrier protein used in the conjugate [4]. The carrier determines the level of induction of specific T cells and therefore the levels of polysaccharide antibodies and hence the protective effect gained by administration of the conjugate vaccine. The optimal carrier might be different for different pneumococcal serotypes [80]. In one study pneumococcal polysaccharide conjugated to a diphtheria carrier was more efficient in inducing a mucosal response, while tetanus conjugate resulted in improved systemic responses [81]. Another study showed that a tetanus conjugate resulted in a better serotype 4 response, while a diphtheria conjugate evoked a better response to types 3, 9 V, and 14 [82].

The addition of adjuvants to conjugate vaccines could potentially reduce the number of doses needed to establish protective immunity and thereby provides protective immunity within a shorter time period and at a reduced cost. Adjuvants also lead to more consistent induction of responses to various polysaccharide serotypes [83]. Toll-like receptor (TLR) ligands have been considered as vaccine adjuvants [84, 85], such as CpG containing oligodeoxynucleotides [86]. Peptide p458 is a peptide derived from the human or mouse 60-kDa heat shock protein (hsp60) and stimulates TLR4 [84]. Conjugated to pneumococcal polysaccharide type 4 it could induce protection in mice against a supralethal *S. pneumoniae* challenge. Protection was associated with polysaccharide type 4-specific IgG antibodies in most but not in all the mice, a T cell response to the p458 carrier and long-term memory. Vaccines composed of p458 conjugated to the polysaccharides of *Salmonella* [87], or meningococcus B and C [88] were also immunogenic in mice, even when injected without an added adjuvant. Other TLR agonists that stimulate TLR8, such as R-848 (TLR7/8), the imidazoquinoline congeners 3M-003 (TLR7/8) and 3M-002 (TLR8), as well as single-stranded viral RNAs (TLR8), also induce a strong immune response in neonates and infants by stimulating p38 MAPK phosphorylation [85]. Furthermore, LT-K63, a nontoxic mutant of *E. coli* heat-labile enterotoxin [89], when administered concomitantly with a conjugated pneumococcal polysaccharide serotype 1, enhanced IgG responses in infant mice compared to conjugated polysaccharide alone [89]. A second dose of conjugated pneumococcal polysaccharide resulted in very high IgG responses and significantly improved protection against lethal pneumococcal infections in this animal model. Similar results were obtained with an MCC vaccine [90].

Furthermore, the route of administration is also shown to be an important determinant in eliciting protective immunity in neonates [4, 89]. Intranasal immunization with conjugated polysaccharides [89, 91] seemed to be effective both in infantile and neonatal mice. A single intranasal dose of conjugate vaccine elicited a sufficient high IgG response to protect neonatal mice against pneumococcal infections, whereas subcutaneous administration required two doses to induce complete protection. The increased efficiency could be explained by the additional induction of a salivary IgA response after intranasal administration. However, antibody responses and protective efficacy remained significantly lower than in adult mice [89]. One of the main reasons for this seems to be the lack of effective adjuvants [92].

Another strategy in early stage of development is the use of surface proteins. An example is the Pneumococcal surface protein A (PspA) that is a cell-wall-associated surface protein [93]. It is known to play a major role in the pneumococcal virulence; it binds human lactoferrin and interferes with complement deposition on the bacterial surface. It is thought that it might result in better immune responses in infants and neonates. The antibody response to PspA has been studied in children [93, 94]. The pneumococcal surface antigen protein A (PsaA) is currently explored as a vaccine candidate. It is structurally conserved [95] and plays a role in adherence to host mucosae [96]. Until now, however, it has not been used as vaccine antigen in humans.

Another potential vaccine strategy is the development of peptides that mimic polysaccharide antigens [97]. The main advantage of using peptides over polysaccharides is that peptides induce a TD antigen response as they are processed by APC’s and presented to T cells. A drawback of the use of peptides in vaccines is their poor chemical stability and subsequently lower immunogenicity in vivo. DNA-based vaccines are another potential approach as they are more stable but were initially not considered a viable option for pathogens coated with polysaccharides since carbohydrate antigens are secondary gene products [97]. However, it was recently shown that a DNAvaccine could induce an IgG2a isotype response against a polysaccharide antigen [98]. Other possible advantages of DNA-vaccines are the relatively straightforward and cheaper production techniques compared to conjugate vaccines.

## 6. CONCLUSION

Neonatal immune responses to polysaccharide pathogens are very weak. Therefore, neonates and young children are at risk for invasive infections with *S. pneumococcus*, *N. meningitidis*, and *H. influenza*. An important percentage of deaths among neonates are caused by these bacteria. The efficacy of the currently used conjugate vaccines is already very high in the population most at risk, but worldwide utilization of these vaccines is hampered by high production costs. Knowledge about neonatal immunological responses to polysaccharide antigens may open the way for the application of newly designed conjugated vaccines or vaccines based on other principles in this patient group. Currently, several strategies are being explored to get insight into the mechanisms underlying the limitations of infant responses and to thereby improve neonatal vaccination efficiency.

## Figures and Tables

**Table 1:** Characteristics of thymus-dependent and thymus-independent antigens

Characteristics	Thymus-dependent	Thymus-independent type 1	Thymus-independent type 2
T_H_-cell activation	++	−	−
IgM-IgG switch	+, IgG1, IgG3	+/−, IgM, IgG2 (low quantities)	−, IgM
Booster response	++	−
Immune response in neonates	High (but lower than in adults)	Intermediate	Low
Development of antibody responses	At birth	3–18 m	24 m
Examples	Protein antigens	LPS	PS
